# Morphology of crystalline–amorphous olefin block copolymers in solution characterized by small-angle neutron scattering and microscopy

**DOI:** 10.1107/S1600576715019226

**Published:** 2015-11-19

**Authors:** Aurel Radulescu, Günter Goerigk, Lewis Fetters, Dieter Richter

**Affiliations:** a Jülich Centre for Neutron Science – Outstation at MLZ, Forschungszentrum Jülich GmbH, Lichtenbergstrasse 1, Garching, 85747, Germany; b Institute of Soft Matter and Functional Materials, Helmholtz Zentrum Berlin für Materialien und Energie, Berlin, 12489, Germany; c School of Chemical and Biomolecular Engineering, Cornell University, Ithaca, NY 14853-5201, USA; d Jülich Centre for Neutron Science (JCNS-1) and Institute for Complex Systems (ICS-1), Forschungszentrum Jülich GmbH, Jülich, 52425, Germany

**Keywords:** block copolymers, crystallization from solution, small-angle neutron scattering, optical microscopy

## Abstract

The single-chain properties and self-assembly behavior in dilute solution of olefin block copolymers obtained by chain-shuttling technology and consisting of alternating crystallizable and amorphous ethylene/1-octene blocks were investigated by pinhole and focusing small-angle neutron scattering techniques, optical microscopy in bright-field and crossed-polarizer modes, and differential scanning calorimetry. The complex hydrocarbon soluble (precipitant free) macro-aggregates formed with decreasing temperature are characterized by spherulitic textures. The spherulites yield, on one hand, a morphology that depends on the chain structure properties and, on the other hand, multiple structural levels with a hierarchical organization that ranges from 10 Å up to tens of micrometres. This morphology displays peculiarities dictated by the polydisperse character of these materials.

## Abstract 2

The multi-level structures and hierarchical morphologies formed by olefin block copolymers in dilute solution are characterized by pinhole and focusing small-angle neutron scattering techniques, optical microscopy in bright-field and crossed-polarizer modes, and differential scanning calorimetry.

## 1. Introduction   

The macroscopic behavior of crystalline–amorphous polymers depends on the constitutive microstructures, which consist of molecules arranged as unit cells, lamellar crystals, fibrils, boards or spherulites (Akpalu, 2010 ▸). These structures can span a wide length scale of several nanometres to hundreds of micrometres. The control and optimization of polymer properties requires the knowledge and understanding of the microstructural properties under various conditions. Recently (Radulescu *et al.*, 2004 ▸, 2006 ▸, 2011 ▸; Radulescu *et al.*, 2008 ▸), we have studied the assembly in solution of a long series of semi-crystalline ethylene-1-butene copolymers (PEB-*n*) with graded ethylene content (where *n* is the number of ethyl branches per 100 backbone C atoms) by combining different small-angle neutron scattering techniques and microscopy. Different architectures of these copolymers have been investigated, *i.e.* PEB-*n* block copolymers with *n* ranging between 7.5 (higher crystallinity) and 11 (lower crystallinity), as well as multi-block copolymers built up by PEB-*n* blocks showing a graded degree of crystallinity. These materials consist basically of microcrystalline ethylene units copolymerized with amorphous butene segments. They exhibit potential as flow improvers for middle distillate refinery fuels as previous structural studies on these wax/PEB-*n* solutions have concluded (Radulescu *et al.*, 2004 ▸; Radulescu, Fetters & Richter, 2012 ▸). Other crystalline–amorphous polymer architectures may also show in solution properties of interest for different applications, particularly as wax crystal modifiers. Recent developments from the DOW Chemical Company have led to the synthesis of novel olefinic block copolymers (OBCs) by using chain shuttling technology (Arriola *et al.*, 2006 ▸). These are ethylene–octene OBCs and jointly consist of crystallizable blocks (hard) with very low octene co-monomer content and high melting temperature alternating with amorphous blocks (soft) with relatively high 1-octene content. These materials are similar in having a statistical multi-block architecture with distributions both in block length and in the number of blocks per chain (Shan & Hazlitt, 2007 ▸). Thus, by varying the hard block content, a broad range of elastomeric and thermal properties emerge. Combined with the ability to form complex nanoscale morphologies, these OBCs enable a wide range of applications to be accessed (Wang *et al.*, 2009 ▸). Owing to the block length polydispersity the OBCs exhibit much larger domain spacing than the traditional block copolymers of similar molecular weight. Unlike the statistical copolymers that form fringed micellar crystals, the OBCs can form space-filling spherulites even for low-crystalline-content material (Wang *et al.*, 2007 ▸). The relationship between the structure and morphology and the crystallization behavior in OBCs, which governs the properties of these materials, remains incompletely explored. Particularly, the assembly properties of these materials in solution have not yet been studied.

Our general aim was to investigate the structural properties of the aggregates yielded in dodecane solution by two low-crystallinity OBCs during the cooling of solutions between the polymer single-coil regime, at high temperatures, and 293 K. The complex morphologies of the OBC aggregates, displaying multiple structural levels spanning a wide length scale, between 10 Å and several micrometres, have been characterized by combining a multitude of techniques: pinhole small-angle neutron scattering (SANS), focusing small-angle neutron scattering (f-SANS), differential scanning calorimetry (DSC) and optical microscopy with crossed polarizers.

## 2. Materials and methods   

**Figure 1:**
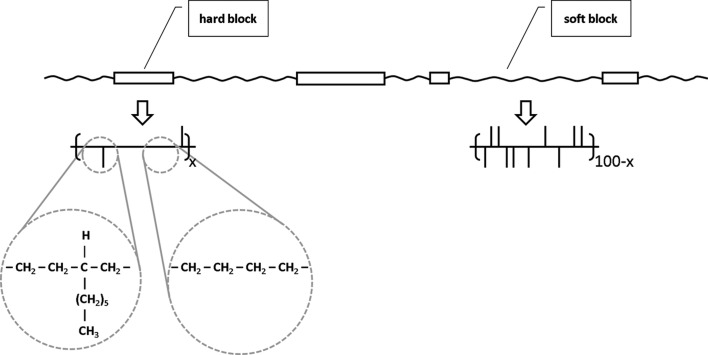
Schematic representation of the OBCs.

Two INFUSE OBCs, 9000 and 9007, were obtained as pellets from the DOW Chemical Company. The overall density of the materials is 0.877 and 0.866 g cm^−3^, respectively, as provided by the manufacturer. These OBCs are ethylene–octene multiblock copolymers (Dias *et al.*, 2008 ▸; Kamdar *et al.*, 2009 ▸) and are shown schematically in Fig. 1 ▸. Polymer solutions were prepared in deuterated dodecane (d-26) at 413 K for polymer volume fractions between φ = 0.1 and 1%. In order to determine the single-coil properties and the self-assembly behavior of the OBCs, the polymer solutions were investigated by SANS over a wide temperature range, between the single-coil regime (>378 K) and the aggregation regime (293–378 K). The 1% polymer solutions at 293 K were additionally investigated by f-SANS.

The SANS measurements were performed at the KWS-2 classical pinhole SANS (Radulescu, Pipich & Ioffe, 2012 ▸; Radulescu, Pipich *et al.*, 2012 ▸) and KWS-3 mirror-focusing f-SANS (Goerigk & Varga, 2011 ▸) diffractometers of the Heinz Maier-Leibnitz Centre (MLZ) at the FRM II reactor in Garching, München (Gläser & Petry, 2000 ▸). In pinhole geometry, using three detection distances (2, 8 and 20 m) and a neutron wavelength λ = 7 Å (Δλ/λ = 20%) at KWS-2, a wavevector transfer *Q* range between 1.5 × 10^−3^ and 0.2 Å^−1^ was covered. At KWS-3, two sample-to-detector distances (SD = 1.5 and 10 m) and the neutron wavelength λ = 12 Å (Δλ/λ = 20%) were used, which allowed us to perform measurements within a *Q* range between 1.2 × 10^−4^ and 0.02 Å^−1^. The special design of the f-SANS instrument, based on the one-to-one mirroring of a small entrance aperture on a high-resolution (0.4 mm) position-sensitive detector by using a double focusing toroidal mirror, enables one to reach such low *Q* values with still sufficient intensity. In both cases, the data were corrected for detector sensitivity, instrumental noise and scattering from the empty cell, then radially averaged and calibrated in absolute units using a Plexiglas standard sample (Radulescu, Pipich *et al.*, 2012 ▸). The results were subsequently corrected for scattering from solvent.

Thus, the combination of the two techniques allowed us to investigate structural sizes from 10 Å to 1 µm. The polymer single-coil characteristics and self-assembly behavior were determined from the investigation of the scattered intensity by SANS at different temperatures within the range from 413 to 293 K, decreasing the temperature in steps of 20 K.

The polymer aggregates in the φ = 1% solution were finally examined using optical microscopy. Solutions dropped on glass lamellae were observed using the bright-field and crossed-polarizer modes with a Leica DM6000M light microscope with polarization options.

Specimens weighing 4 mg were cut from the original polymer pellets for a supplementary thermal analysis on a PerkinElmer Series 7 differential scanning calorimeter. Scans were taken under a nitrogen atmosphere between 10 and 423 K with a heating/cooling rate of 5 K min^−1^.

Additional insight about the morphology and substructure of polymer assemblies was obtained by the examination of the wax decorated polymer aggregates under the microscope, a method used before for the indirect visualization of PEB-7.5 polymeric needles (Radulescu *et al.*, 2006 ▸). Polymer–wax mixed assemblies formed upon cooling from 413 K in common dodecane solution of OBCs with 1 and 4% hexatriacontane wax (C_36_). These were complementarily examined at 293 K, below the wax precipitation point (Radulescu *et al.*, 2008 ▸), with the Leica DM6000M light microscope in bright-field mode.

## 3. Results and discussion   

### 3.1. Thermal behavior   

**Figure 2:**
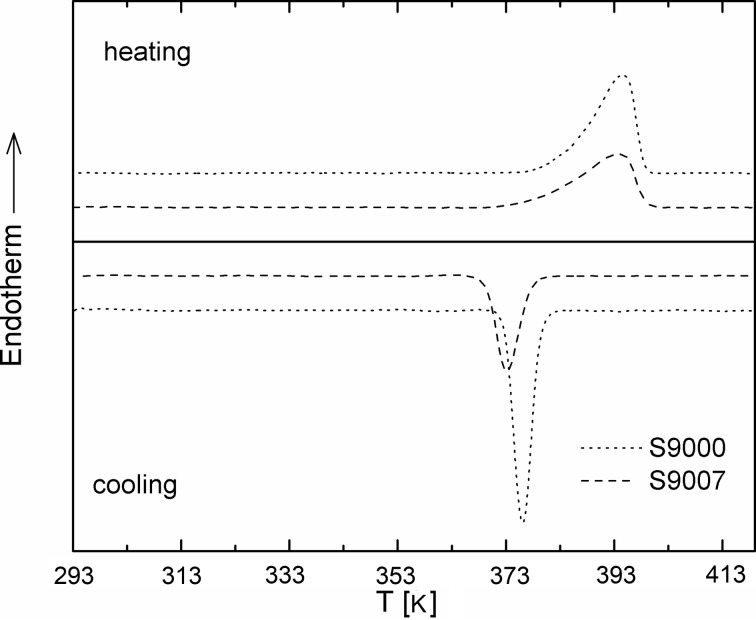
Thermal behavior of OBCs: the melting (top) and crystallization (bottom) processes at a rate of 5 K min^−1^.

The DSC curves of the melting and the subsequent crystallization of the two OBCs are shown in Fig. 2 ▸. The values of melting temperature (*T*_m_) and crystallization temperature (*T*_c_) are summarized in Table 1 ▸. The high melting temperature (around 393 K for both OBCs) is typical for the OBCs in which the long ethylene sequences of the hard blocks crystallize as large, chain-folded lamellae (Wang *et al.*, 2009 ▸). Although the position of the melting peak is similar for the two OBCs, the heat of melting taken as the area of the peaks differs sizably. First insights about the crystallinity from heat of melting *X*_c,Δ*H*_ of the two materials (total hard and soft blocks) were obtained from the direct interpretation of the crystallization peaks following a procedure described elsewhere (Wang *et al.*, 2009 ▸). The 9000 OBC presents a higher crystallinity than the 9007 OBC, which leads to the conclusion that the global co-monomer content (1-octene) in the 9007 OBC is higher than in the 9000 OBC. This observation is strengthened by the crystallization behavior of the two OBCs, which shows sizable differences both in the position and in the area of the crystallization peaks.

### 3.2. Optical microscopy observations   

**Figure 3:**
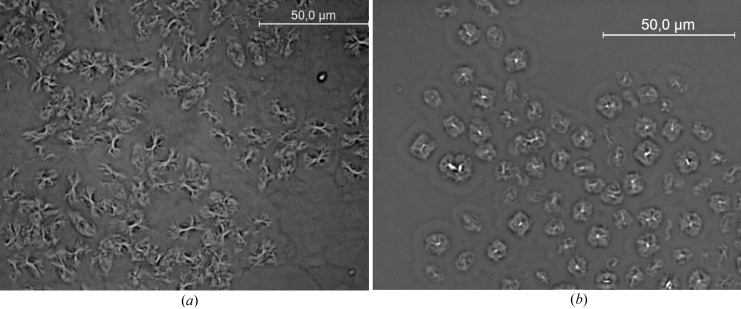
Micrographs of the assemblies of 9000 (*a*) and 9007 (*b*) OBCs in dodecane observed at 293 K using bright-field microscopy.

**Figure 4:**
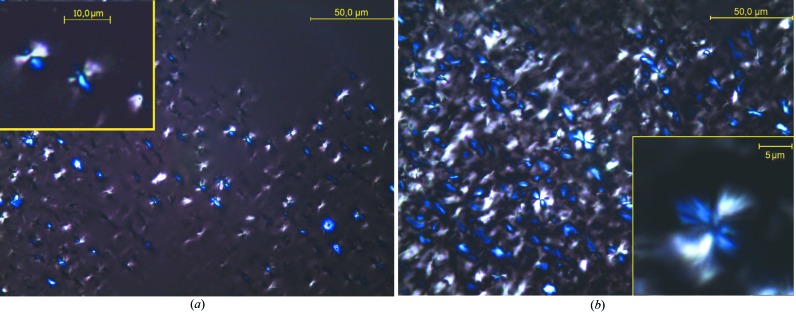
Crossed-polarized micrographs of the assemblies of 9000 (*a*) and 9007 (*b*) OBCs in dodecane at 293 K.

Fig. 3 ▸ shows images of typical aggregates formed by the two OBCs (φ = 1%). These were collected at 293 K using the bright-field observation mode. Although in both cases large aggregates with sizes of about 10 µm are observed, a detailed inspection of the micrographs reveals that the morphologies yielded by the two OBCs in solution differ. In the case of the 9000 OBC, aggregates resembling bundles or sheaves of elongated objects are clearly revealed. The 9007 OBC gives rise to spherical aggregates that show a dense nucleus. Observed between crossed polarizers (Fig. 4 ▸), both types of macro-aggregates exhibit dark and bright patterns due to birefringence (the ‘Maltese cross’ feature). This shows that both OBCs can yield in solution crystalline–amorphous spherulites. Typically, a polymer spherulite represents a spheriform cluster of fibrillar- or lamellar-like crystallites alternating with amorphous domains which start growing from a nucleation site and splay out laterally (Magil, 2001 ▸; Gránásy *et al.*, 2005 ▸). Both types of polymer assembly show a hierarchical structural organization.

**Figure 5:**
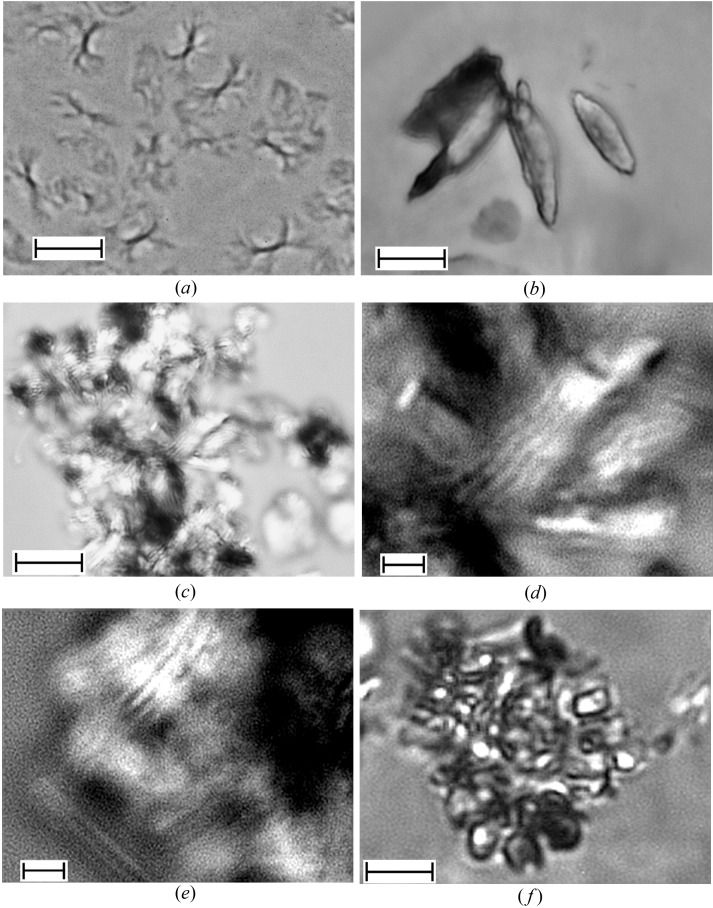
Micrographs of the polymer assemblies and polymer–wax common aggregates in deodecane observed at 293 K using bright-field microscopy for the 9000 OBC (*a*), the 9000 OBC and 1% C_36_ wax (*b*), the 9000 OBC and 4% C_36_ wax (*c*)–(*e*), and the 9007 OBC and 1% C_36_ wax (*f*); scale bar: 10 µm (*a*)–(*c*), (*f*), 2 µm (*d*), (*e*).

For the case of the 9000 OBC such a feature is evident by examining the micrographs under higher magnification (Fig. 5 ▸ *a*). The sheaf-like morphology exhibits in this case fibrillar-like structural sub-units: a central trunk with a thickness of about 1 µm, possibly consisting of a bundle of fibrils, which shows at both ends sub-micrometric scattered branches. For the more homogeneous aggregates formed by the 9007 OBC no clear features were revealed by higher magnifications and the substructure may be only presumed on the basis of the structural peculiarities typically exhibited by the crystalline–amorphous spherulites.

An attempt to observe the substructures of the spherulitic morphology by transmission electron microscopy (TEM) was also made. No obvious morphology or structure was observed by TEM. It seems that either the macro-aggregates observed by optical microscopy in dodecane solutions were easily destroyed by the procedure of the sample preparation for TEM observation or the substructure in the spherulitic morphologies cannot be recognized by TEM because they are thicker and visualized through a projection of too many structural elements which averages out all structures in the images.

The substructure of the OBCs’ spherulites was highlighted indirectly by decorating the polymeric aggregates with wax crystals formed at lower temperature than the polymer crystallization temperature.

When the 9000 OBC is mixed in common solution with a small wax volume fraction of C_36_ hexatriacontane (φ_wax_ = 1%), the resulting system has a wax crystallization point much lower than the sole OBC’s crystallization temperature. Here, small spindle-like wax structures were formed (Fig. 5 ▸ *b*). This crystallization habit differs markedly from the typical ‘house-of-cards’ wax morphologies several hundreds of micrometres in size (Radulescu *et al.*, 2008 ▸) and seems to be a consequence of the wax nucleation and controlled growth onto the polymer sheaf-like overall morphology (Fig. 5 ▸ *a*). In the case of higher wax contents (φ_wax_ = 4%), *i.e.* higher wax crystallization point, larger agglomerates are formed (Fig. 5 ▸ *c*). Higher magnification (Fig. 5 ▸ *d* and 5 ▸ *e*) reveals that the agglomerates consist of aligned arrangements of parallel needle-like aggregates. The needles are about 10 µm long and, taking into account possible off-focusing effects that are inherent at this magnification power, have a thickness of 0.5 µm or smaller. The correlation distance between needles seems to be also smaller than 0.5 µm. Finally, macro-aggregates consisting of agglomerations of smaller platelet-like structures can be observed in the common solution of 9007 OBC and C_36_ wax for φ_wax_ = 1% (Fig. 5 ▸ *f*). The formation of such a morphology is again a consequence of the crystallization of wax molecules templated by initial polymer aggregates, which in this case appear to be lamellar spherulites.

### 3.3. Small-angle neutron scattering   

**Figure 6:**
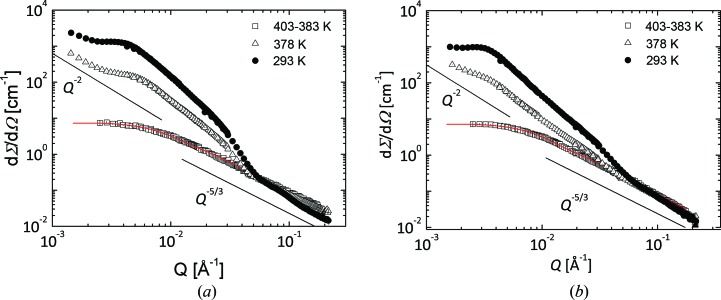
Small-angle neutron scattering cross sections from solutions of 9000 (*a*) and 9007 (*b*) OBCs in d-26 (φ = 1%) measured at different temperatures. The solid lines indicate the power-law behavior in different *Q* ranges, whereas the red curves represent the model description of the single-coil polymer morphologies (see the discussion in text).

Figs. 6 ▸(*a*) and 6 ▸(*b*) display the scattering profiles from the 1% polymer solutions measured at different temperatures by SANS. The power law exponents (*Q* ^−*p*^) specific for different structures formed by the copolymers are given. Hence, swollen single coils (*p* = 5/3), one-dimensional (*p* = 1) and platelet-like (*p* = 2) morphologies, or smooth interfaces (*p* = 4) can be identified and characterized (Radulescu *et al.*, 2011 ▸). The model curves of the single-coil polymer morphologies described later are also depicted.

Within the temperature range from 413 to 283 K all polymers are dissolved as single coils, as shown by the single-chain form factor features identified in the scattering profiles. The plateau towards low *Q* yields information about the volume fraction and molecular weight. The bending down of the intensity is characteristic of the Guinier regime, from which information about the radius of gyration of the polymer coil is gleaned. The *Q* ^−5/3^ power law regime towards high *Q* is indicative of excluded volume interactions between the chain segments.

Characterization of the single-chain properties was possible from the analysis of the data measured for different polymer concentrations in terms of the Zimm approximation:
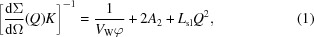
where *K* = *N*_A_/Δρ^2^ is the inverse contrast factor (*N*_A_ being the Avagadro constant and Δρ^2^ being the contrast factor between the polymer and the solvent), *V*_W_ is the molar volume, *A*_2_ is the second virial coefficient and *L*_sl_ = *R*_g_^2^/(3φ*V*_W_), with φ and *R*_g_ the polymer volume fraction and radius of gyration, respectively.

**Figure 7:**
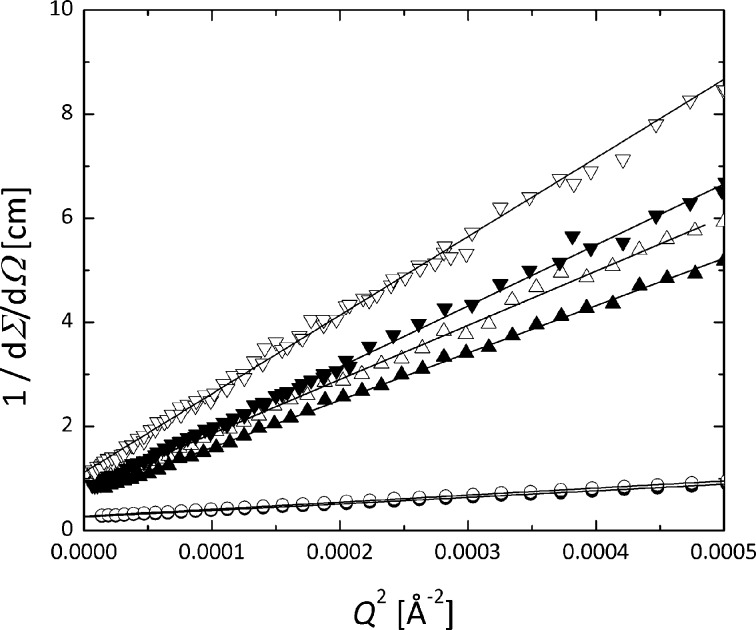
Zimm plot of the scattering from single chains of 9000 (filled symbols) and 9007 (open symbols) OBCs in d-26 at 403 K, for different polymer concentrations (φ = 1, 0.2 and 0.1% from bottom to top).

This analysis (Fig. 7 ▸) results in a radius of gyration *R*_g_ of 195 (14) Å for the 9000 OBC and 196 (18) Å for the 9007 OBC. The analysis of the ‘forward scattering’ dΣ/dΩ(0) as a function of polymer volume fraction resulted in the molar volume *V*_W_ which delivered the molecular weights (*M*_W_) of 158.9 ± 21.8 kg mol^−1^ and 116.6 ± 20.4 kg mol^−1^ for the two OBCs. Typically, these INFUSE materials contain chains of two to ten alternating blocks, while the hard block weight average molecular weights range from 7 to 28 kg mol^−1^ (Wang *et al.*, 2009 ▸; Kamdar *et al.*, 2009 ▸). An overall fit of the single-chain scattering patterns with the Beaucage form factor (Beaucage, 1996 ▸) is possible:
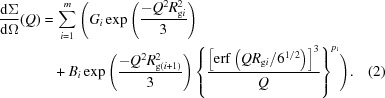
For a single structural level (*m* = 1) the use of the parameters from the Zimm analysis (the ‘forward scattering’ *G* and *R*_g_) and an exponent *p* = 5/3 indicative of swollen single chains was successful, as one can see in Fig. 6 ▸. Following the assumption of a ‘polymeric constraint’ (Beaucage, 1996 ▸; Hammouda, 2010 ▸) the parameter *B* in equation (2) was expressed in terms of *G*, *R*_g_ and *p*, hence reducing the number of free parameters.

A decrease in the temperature resulted in the formation and evolution of polymer aggregates that led to an increase of the scattering level towards low *Q* compared to the single-chain scattering feature. For both OBCs the first aggregation indications were observed in the scattering data at 378 K. The scattered intensity increases monotonically up to 293 K (not shown in Fig. 6 ▸), indicating that the aggregates grow in size and number.

The scattering patterns are characterized by power law regimes with characteristic exponents for the morphologies formed.

The self-assembly behavior of the two OBCs at the length scale between 10 and 1000 Å (SANS range) is very similar. The scattering profiles from both materials in the aggregation regime (Figs. 6 ▸) display the same peculiarities: a seemingly *Q* ^−2^ power law behavior of the scattered intensity and a peak-like feature in the low-*Q* range of the SANS domain.

The *Q* ^−2^ power law indicates the formation of two-dimensional lamellar-like aggregates. The peak-like feature may denote either a large interlamellar domain spacing or the presence of a higher-order structure. Higher-order scattering maxima are not usually seen because of the washing out of the fine structure resulting from the distribution of spherulite size or disorder. From the analysis of the microscopy images and the TEM attempts no peculiarities of higher-order structure were observed. On the other hand, it is known that, in block copolymers, increasing polydispersity is expected to lead to substantially increased domain periodicity compared with near-monodisperse block copolymers having similar chemical composition. The interlamellar domain spacing can be calculated according to D* = 2π/Q*, where Q* represents the peak position. These characteristics are typical for a morphology made of crystalline lamellae alternating with amorphous interlamellar regions similar to that reported in the case of crystallization of OBCs from the melt (Wang *et al.*, 2007 ▸, 2009 ▸). With decreasing temperature a weak shift of the peak position towards lower *Q* values is observed, which indicates an increase of the interlamellar spacing. The estimated interlamellar distances at 293 K, D* ≃ 1700 Å and D* ≃ 2300 Å for the 9000 and 9007 OBCs, respectively, are very large, untypical for traditional block copolymers of similar mol­ecular weights (Green, 2001 ▸).

**Figure 8:**
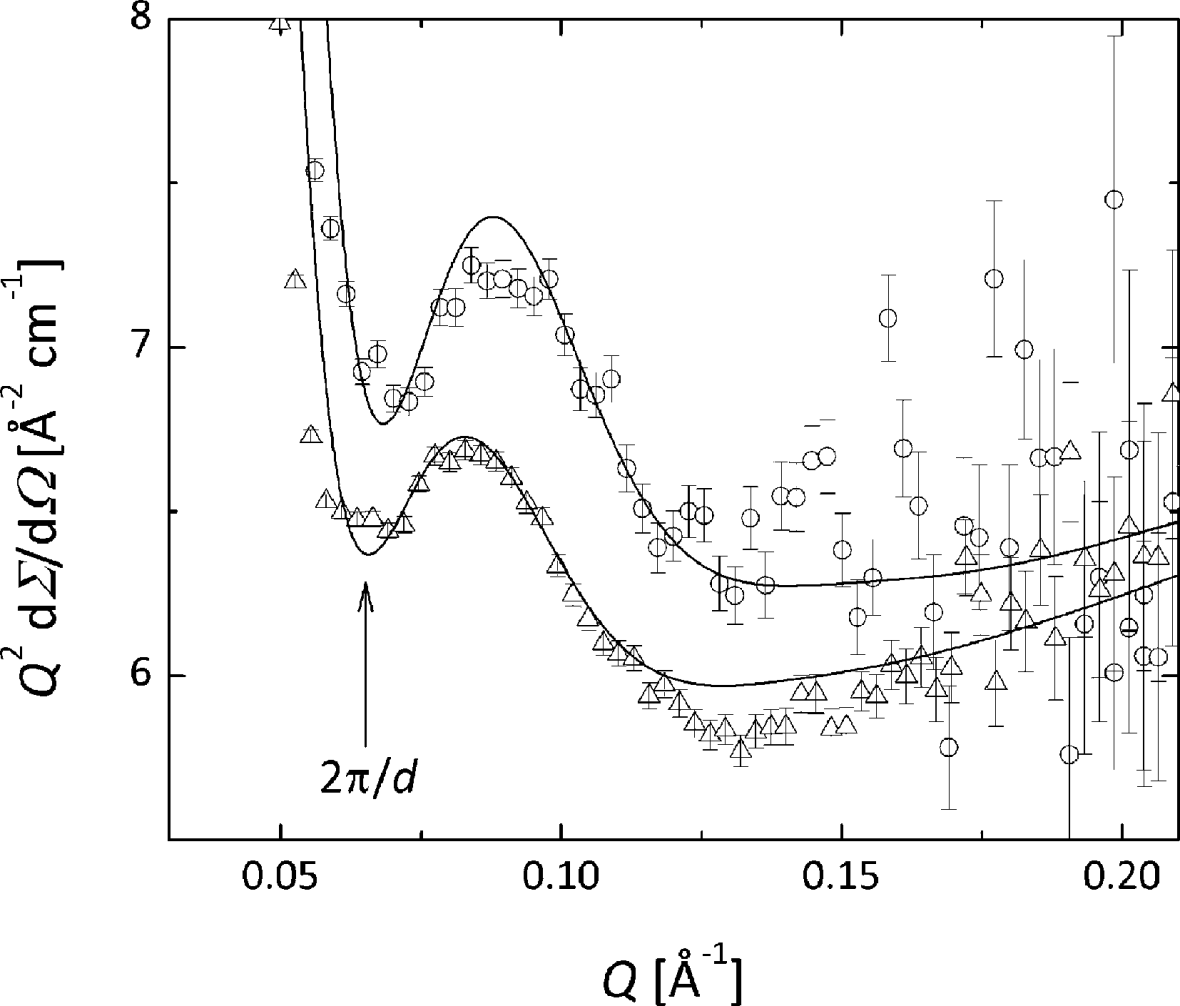
Kratky plot of the scattering in the high-*Q* region from the assemblies of 9000 (open triangles) and 9007 (open circles) OBCs in d-26 at 293 K (φ = 1%); the curves represent the model fit of the polymer morphologies (see text).

On the other hand, the bending down of the scattering patterns at 293 K observable at *Q* ≃ 0.05 Å^−1^ and the weak hump-like structural feature noticeable towards high *Q* resemble the form factor details of a much thinner density profile perpendicular to the lamellar surface, which seems to correspond to the thickness of the crystalline lamellar core. From the presentation of the scattering data in a Kratky-type plot (Fig. 8 ▸) the thickness *d* of the crystalline lamellae can be extracted in a direct fashion through *Q*_min_ = 2π/*d*.

Using this crude approach a lamellar thickness of about 100–110 Å is roughly estimated for both OBCs. This very low value compared to D* indicates that either the amorphous interlamellar regions are very thick or crystalline–amorphous morphologies well separated from one another are kept together in a correlated arrangement (stack) by a special mechanism. The combined SANS and microscopy findings lead to the conclusion that the lamellar stacks may consist of correlated thin elongated crystalline lamellae surrounded on both faces by amorphous layers (core–brush morphology), which are well separated from each other by regions characterized by very low polymer content.

Such morphology would offer in a common solution with wax molecules a nucleation platform for wax crystal formation at low temperature, on one hand, and a control mechanism of a limited wax crystal growth through the polymer brush within the amorphous region, on the other hand. The mechanism would be similar to that observed in the case of wax crystallization on polyethylene-poly(ethylene-propylene) PE-PEP (Richter *et al.*, 1996 ▸) or PEB-7.5 (Radulescu *et al.*, 2004 ▸) crystalline–amorphous two-dimensional morphologies. Wax crystal morphologies such as those observed in Figs. 5 ▸(*b*)–5 ▸(*f*) can be explained in this case by the templation and control of wax crystal growth *via* peculiarities of the primordial polymer aggregates.

**Figure 9:**
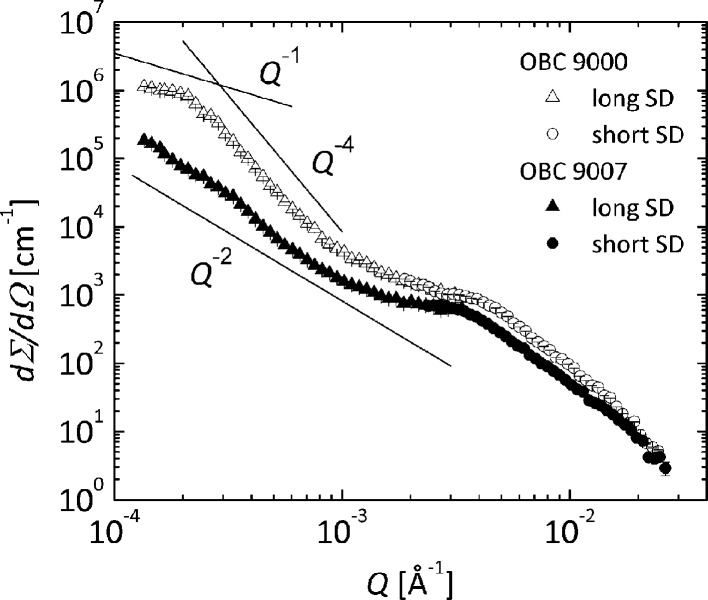
Small-angle neutron scattering cross sections from solutions of 9000 and 9007 OBCs in d-26 (φ = 1%) measured at 293 K using f-SANS. The solid lines indicate the power-law behavior in the low *Q* range. Data collected at two sample-to-detector (SD) distances are shown.

Fig. 9 ▸ shows the f-SANS scattering profiles from the two OBCs at 293 K. Although the peak-like features are smoother owing to the resolution, the data collected at the short detection distance are similar to the low-*Q* data measured by conventional pinhole SANS. The very low *Q* patterns are significantly different for the two polymers. The *Q* ^−2^ power law in the case of the 9007 OBC extends down to the lowest *Q* value, displaying a kind of shoulder, which suggests a bimodal size distribution. The observed shoulder at *Q* ≃ 0.00035 Å^−1^ could be indicative of the thickness of a lamellar stack. The extended *Q* ^−2^ behavior indicates that the lamellar morphologies show lateral extensions up to 5000 nm, thereby covering a length scale comparable to the radius of the spherulites observed by optical microscopy (Fig. 3 ▸ *b*).

In contrast, the 9000 OBC scattering pattern exhibits towards low *Q* a steep power law (*p* = 4) and terminates towards the lowest *Q* in a Guinier-like regime. Here, the commencement of an apparent *Q* ^−1^ power law and thus the formation of the large-scale fibrillar-like structures revealed by the optical microscopy observations may be presumed. An analysis of the low-*Q* data in terms of the one-dimensional Guinier approximation (Schwahn *et al.*, 2002 ▸) revealed a rod (fibril) thickness of about *a* = 1.5 µm, which is quite consistent with the optical microscopy observations.

**Figure 10:**
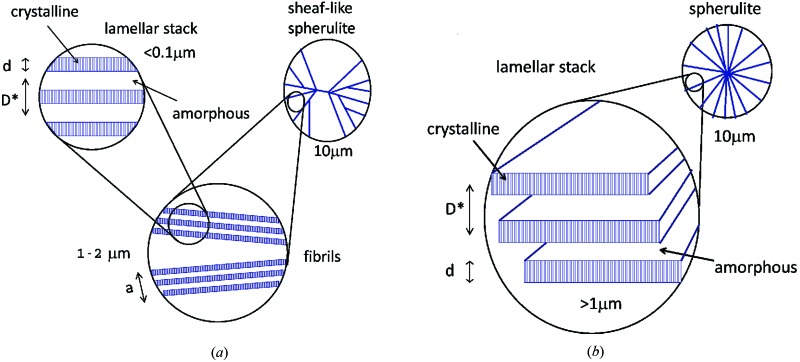
The hierarchical organization of the main structural levels displayed by the macro-aggregates formed by the 9000 (*a*) and 9007 (*b*) OBCs in solution, as emerged following the interpretation of the scattering data and DSC and microscopy observations.

On the basis of the qualitative information delivered by DSC, microscopy and wide-*Q*-range SANS a hierarchical morphology of the macro-aggregates formed by the two OBCs in solution is schematically proposed in Fig. 10 ▸. The aggregates show multiple structural levels and a hierarchical organization on a length scale from 10 Å up to tens of micrometres. In the case of the OBC with higher crystallinity (the 9000 OBC) a crystalline–amorphous primary morphology consisting of thin lamellar slabs (core–brush morphology) arranged in stacks and characterized by a large interlamellar spacing was formed. The correlated lamellae grow mostly longitudinally, exhibiting at a larger length scale a board-like or one-dimensional overall aspect (Schmidt-Rohr, 2007 ▸). These are the nucleation platforms for crystallization of wax molecules from common polymer–wax solutions, which template the correlated arrangements of parallel needle-like structures observed in Figs. 5 ▸(*d*) and 5 ▸(*e*). A lamellar crystal is typically formed as a result of the tendency of crystallizable segments to attach progressively to a preferred growth plane. Upon cooling of the OBCs, the crystallization of the hard blocks forces the segregation of the non-crystallizable blocks into the interlamellar regions (Wang *et al.*, 2009 ▸). A good separation of the hard block lamellar crystals from the interlamellar soft blocks appears to be present even in the copolymers with very low hard block content (Wang *et al.*, 2007 ▸).

The combination of SANS and microscopy offered a unique method for a complete understanding of the complex morphology in the case of the 9000 OBC: the thin crystalline lamellae surrounded on both sides by amorphous layers are separated from each other by a third layer of a very low material content. The stacked elongated lamellae of the 9000 OBC give rise to a secondary fibril-like morphology with a lateral size within the micrometre scale. Wax crystals formed at high temperature (high wax content) decorate this morphology and emphasize it under the optical microscope (Fig. 5 ▸ *d* and 5 ▸ *e*). The fibrils associate in bundles and branch, leading to the formation of the tertiary morphology represented by the sheaf-like spherulites observed in Fig. 5 ▸(*a*). Crystallization of wax molecules at low temperature (low wax content) on the skeleton of such polymeric structures gives rise to the formation of spindle-like crystals (Fig. 5 ▸ *b*).

In the case of the 9007 OBC (the material with lower crystallinity) correlated lamellae with a very large lateral extension apparently grow from nucleation sites and splay out in fan-like spherulitic morphologies, dominated by large amorphous regions. This is the typical case when a spherulitic morphology results from the pressure of non-crystallized segments emerging from the lamellar surfaces, which impose the divergent arrangement of the lamellae. This can explain the more regular and homogeneous aspect of the macro-aggregates observed by bright-field microscopy (Fig. 3 ▸ *b*), which dictates also the peculiarities of the wax crystallization from a common polymer–wax solution (Fig. 5 ▸ *f*).

Although both OBCs show similar structural features at the sub-micrometre length scale, their structural features at the micrometre length scale and the overall aspects (Fig. 10 ▸) are very different. This behavior appears to be a consequence of the chain characteristics of the two polymers (Fig. 1 ▸). The material with higher 1-octene content (longer sequences with side groups), 9007 OBC, yields the more regular and homogeneous morphologies, with preponderant amorphous behavior. The large platelets schematically depicted in Fig. 10 ▸(*b*) should be seen as isolated lamellae surrounded on both sides by broad amorphous phases. It is worth noticing (Wang *et al.*, 2007 ▸) that the lamellae have a short and discontinuous character. Conversely, the formation of irregular bundle- and sheaf-like spherulitic morphologies is favored by the 9000 OBC sample (longer and/or frequent crystallizable sections in Fig. 1 ▸). Typically, the sheaf-like type of spherulite is thought to be the result of homogeneous nucleation when a single needle is formed and subsequently branches. The 9000 OBC morphology seems to arise as a consequence of homogeneous nucleation and growth of fibril-like structures accompanied or followed by frequent co-crystallization and cross-linking events occurring between these structures.

A quantitative characterization of the lamellar stacks was done by fitting the SANS data (*Q* > 0.001 Å^−1^) in terms of the structural model of correlated crystalline lamellae surrounded on both sides by amorphous layers coexisting with single chains in solution. This approach was successfully used for the characterization of the lamellar assemblies of PE-PEP crystalline–amorphous diblock copolymers (Richter *et al.*, 1996 ▸) and the lamellar crystals of PE (Wang, 2004 ▸) in solution. The model combines the form factor of individual crystalline–amorphous lamellar slabs (core–brush morphology) with the paracrystalline structure factor describing the stacking effects [equation (3)] over distances larger than the thickness of the lamellar slabs:
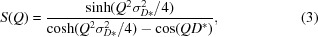
with σ_D__*_ the smearing parameter of D*. As long as the lateral extension of lamellae and lamellar thickness are well separated in length scale, which is the case for the examination of the SANS data, the scattering cross section may be expressed as
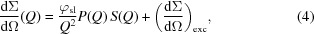
where φ_sl_ is the volume fraction of lamellar slabs, *P*(*Q*) the form factor of a polymer crystalline–amorphous lamellar slab (Richter *et al.*, 1996 ▸; Wang, 2004 ▸) and *S*(*Q*) the structure factor of stacking slabs. An excess scattering which may originate from polymer blob structures (due to excluded volume interactions between chains in the amorphous brush-like region) or from polymers that are still in solution in a single-coil conformation is added. This is an important contribution to the scattering intensity at high *Q* and is described by the Beaucage form factor [equation (2)] for *p* = 5/3.

The lamellar slabs consist of crystalline cores of thickness *d* surrounded on both sides by amorphous brushes of thickness *l*_b_ which are separated by a distance D* that, according to an above-mentioned assumption, fulfills the condition D* >> *d* + 2*l*_b_. In a very simple approach, for both crystalline and amorphous layers a homogeneous rectangular density profile characterized by the corresponding contrast factors (Δρ_c_)^2^ and (Δρ_b_)^2^ is considered. The contrast factors account for the difference between the scattering length density of the solvent ρ_0_ and that of the polymer ρ_p_ multiplied with the polymer volume fraction inside the crystalline (φ_c_) and amorphous regions (φ_b_), respectively. The form factor *P*(*Q*) of the density profile along the direction perpendicular to the faces of the lamellar slab contains the contrast factors squared, which include the volume fractions of polymer φ_b_ and φ_c_ within the amorphous and crystalline layers, respectively (φ_c_ = 1 is assumed).

**Figure 11:**
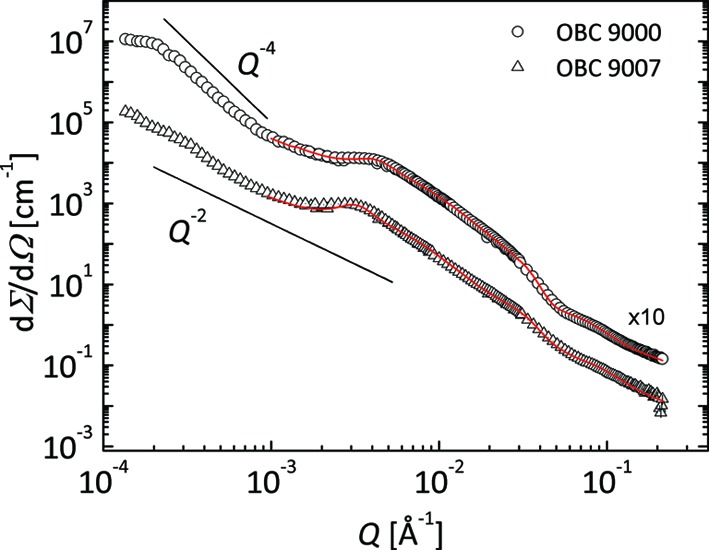
The experimental (symbols) and modeled (lines) small-angle neutron scattering cross sections from solutions of 9000 and 9007 OBCs in d-26 (φ = 1%) at 293 K; for clarity the data from OBC 9000 are shifted vertically by a factor of ten.

Thus, the fitting procedure assumes a large number of free parameters (*d*, *l*_b_, D*, σ_D*_, φ_sl_, φ_b_, *R*_g_^blob^, *G* ^blob^) that must be determined in order to acquire a meaningful characterization of the complex morphology. In order to get reliable fits and values for the free structural and density parameters the modeling of the whole SANS pattern was done in a two-step procedure. In the initial step, which assumed only the fit of the high-*Q* data, with the benefit of the data analysis in the Kratky approach (Fig. 8 ▸) the crystal thicknesses *d* = 110 (15) and 93 (17) Å and the high-*Q* excess scattering for the 9000 and 9007 OBCs were determined in a straightforward way. In the second step the crystalline–amorphous morphology was modeled using equation (4) with a reduced number of free parameters. A good fit of the whole scattering pattern for a reasonable set of values of the geometrical and density parameters is depicted in Fig. 11 ▸. The main parameters obtained in this case are D* = 1750 Å, σ_D*_ = 720 Å, *l*_b_ = 180 Å, φ_b_ = 0.12 for the 9000 OBC and D* = 2150 Å, σ_D*_ = 870 Å, *l*_b_ = 220 Å, φ_b_ = 0.30 for the 9007 OBC.

The fitting approach represents a simplification of a more realistic case assuming a specific polymer density profile inside the amorphous region. This case, requiring a higher number of free parameters and thus a complicated and less reliable fitting procedure, was not considered here. Qualitatively, the results of the fitting are consistent with the chain characteristics. The 9007 OBC, the polymer with the lower crystallinity and thus large soft block content, presents a larger thickness of the amorphous layer and a higher polymer volume fraction in the amorphous region than the more crystalline 9000 OBC. The latter yields thicker crystalline lamellae and more compact macro-aggregates. Also, it is worth noting that the needle-like wax structures show a thickness of about 0.5 µm (Figs. 5 ▸ *d* and 5 ▸ *e*), which is in good agreement with the total thickness of the core–brush morphology of 9000 OBC, and this morphology presumably templates the final wax structure.

The main morphological characteristics obtained by SANS, namely the unusually large interlamellar distance and the peculiarity that the crystalline–amorphous lamellae are well separated, are similar to those observed for the morphology of OBCs crystallized from the melt. There, much larger interlamellar spacings than expected for traditional block copolymers of similar *M*_W_ were reported (Hustad *et al.*, 2009 ▸; Jin *et al.*, 2010 ▸). In these cases, the large interlamellar spacing was considered to be a direct consequence of the polydisperse character of the OBCs, which causes anomalously thick amorphous regions. It was observed that in melts, following perturbations occurring during the sample preparation, a fraction of the molecules with a large mismatch in block length are driven away from the lamellar interface and swell the domain preferred by the longer blocks (Hustad *et al.*, 2009 ▸). It was also observed that more amorphous phases are rejected outside of the lamellar stacks (Tong *et al.*, 2012 ▸). Thus, it was demonstrated (Jin *et al.*, 2010 ▸) that smaller perturbations favor the expelling of unbalanced molecules for which the entropy lost by the longer block opposes the enthalpy gained by surrounding the short blocks with similar segments. In the case of the morphologies formed by the OBCs in solution a similar behavior might also be responsible for the observed large separation of the lamellar slabs. The high polydispersity degree (both in block length and in the number of blocks per chain) and relatively low crystallinity would favor the formation of broad amorphous phases and narrow crystalline layers. The increase of the interlamellar spacing in time or with lowering temperature would be a consequence of the gradual swelling of the amorphous phase based on the continuous expelling of unbalanced molecules, which ultimately will return in the solution.

Finally, as already demonstrated by the incipient optical microscopy observations of mixed polymer–wax aggregates, the crystalline–amorphous OBC assemblies may represent an appropriate environment for dictating the habit and controlling the size of the waxy crystals grown in hydrocarbon solutions. The usefulness of the OBCs in such applications will be the subject of a future contrast-matching SANS structural study on ternary polymer–wax–solvent systems.

## 4. Conclusions   

The chain properties and the self-assembly behavior of two olefin block copolymers obtained by chain-shuttling chemistry were investigated by combined wide-*Q*-range small-angle neutron scattering, optical microscopy with crossed polarizers and DSC. The complex morphologies yielded with decreasing temperature multiple structural levels that are hierarchically organized and with a size range from 10 Å to tens of micrometres. The peculiarities observed in the structure and morphology of the OBC macro-aggregates are a consequence of the chain characteristics and of the polydispersity in block length and in the number of blocks per chain. These self-assembling multi-block polymers show the formation of soluble complex hydrocarbon architectures that are converted to single coils above ∼378 K. This reversible behavior suggests that the INFUSE block copolymers can have applications as downstream petroleum additives. These could include uses such as wax crystal and viscosity index modifiers.

## Figures and Tables

**Table 1:** Characteristics and physical properties of OBCs

OBC	Density† (g cm^−3^)	*T*_m_ (K)	*T*_c_ (K)	*X*_c,Δ*H*_ ‡ (wt%)	*M*_W_ § (kg mol^−1^)	*R*_g_ § (Å)
9000	0.877	394.6	376.2	12	159	195
9007	0.866	393.8	373.2	8	117	196

† Density values taken from the manufacturer data sheets.

‡ Crystallinity determined by DSC.

§ Chain characteristics determined by SANS.
